# Prevalence and infection risk factors of bovine *Eimeria* in China: a systematic review and meta-analysis

**DOI:** 10.1051/parasite/2021055

**Published:** 2021-08-10

**Authors:** Dong-Li Li, Qing-Long Gong, Gui-Yang Ge, Qi Wang, Chen-Yan Sheng, Bao-Yi Ma, Zi-Yang Chen, Yang Yang, Jian-Ming Li, Kun Shi, Xue Leng, Rui Du

**Affiliations:** 1 College of Chinese Medicine Materials, Jilin Agricultural University Changchun Jilin Province 130118 PR China; 2 College of Animal Science and Technology, Jilin Agricultural University Changchun Jilin Province 130118 PR China; 3 Laboratory of Production and Product Application of Sika Deer of Jilin Province, Key Laboratory of Animal Production, Product Quality and Security, Ministry of Education, Jilin Agricultural University Changchun Jilin Province 130118 PR China

**Keywords:** *Eimeria*, Cattle, Mainland China, Prevalence, Meta-analysis

## Abstract

*Eimeria* spp. cause the disease coccidiosis, which results in chronic wasting of livestock and can lead to the death of the animal. The disease, common worldwide, has caused huge economic losses to the cattle industry in particular. This is the first systematic review and meta-analysis of the prevalence of bovine *Eimeria* in China. Our search of five databases including PubMed, ScienceDirect, China National Knowledge Infrastructure (CNKI), Chongqing VIP, and Wan Fang for articles published up to February 29, 2020 on the prevalence of *Eimeria* in cattle in mainland China yielded 46 articles, in which the prevalence of cattle ranged from 4.6% to 87.5%. The rate of bovine *Eimeria* infection has been decreasing year by year, from 57.9% before 2000 to 25.0% after 2015, but it is still high. We also analyzed the region, sampling years, detection methods, feeding model, seasons, and species of bovine *Eimeria*. We recommend that prevention strategies should focus on strengthening detection of *Eimeria* in calves in the intensive farming model.

## Abstract (fr)

Les espèces d’*Eimeria* provoquent la coccidiose, une maladie qui entraîne l’émaciation chronique du bétail et peut entraîner la mort de l’animal. La maladie, répandue dans le monde entier, a causé d’énormes pertes économiques à l’industrie bovine en particulier. Ceci est la première revue systématique et méta-analyse de la prévalence des *Eimeria* des bovins en Chine. Notre recherche dans cinq bases de données, dont PubMed, ScienceDirect, China National Knowledge Infrastructure (CNKI), Chongqing VIP et Wan Fang, pour des articles publiés jusqu’au 29 février 2020, sur la prévalence des *Eimeria* chez les bovins en Chine continentale, a donné 46 articles, dans lesquels la prévalence chez les bovins variait de 4,6 % à 87,5 %. Le taux d’infection des bovins par *Eimeria* a diminué d’année en année, passant de 57,9 % avant 2000 à 25,0 % après 2015, mais il est toujours élevé. Nous avons également analysé la région, les années d’échantillonnage, les méthodes de détection, le modèle d’alimentation, les saisons et les espèces d’*Eimeria* de bovins. Nous recommandons que les stratégies de prévention se concentrent sur le renforcement de la détection des *Eimeria* chez les veaux dans les élevages intensifs.

## Introduction

Eimeriosis is caused by protozoa of the phylum Apicomplexa, family Eimeriidae and genus *Eimeria*, one of the most common parasitoses in cattle throughout the world [9]. *Eimeria* live in the intestinal cells of infected cattle [5]. The biological cycles of *Eimeria* are complex; the infectious form of *Eimeria* (sporulated oocysts) spreads rapidly over soil, vegetation, or water, and it can survive in these environments for several months [7]. A few *Eimeria* spp. can cause clinical symptoms, such as diarrhea and weight loss. [29]. The disease occurs seasonally. *Eimeria* oocysts develop faster in a humid environment, and it is easy for cattle to be infected when the feed composition changes suddenly, the animals are already suffering from infectious disease, or other factors have caused decreased resistance [76]. *Eimeria* can infect various breeds of cattle. Infection is more common in calves less than one year old, and adult cattle often play the role of carrier. Generally, cattle will be infected with at least two species of *Eimeria* at the same time, and as many as eight species have been detected in a single sample [93]. In about 70% of calves that are infected with *Eimeria*, there are two or more species of the parasite [68]. The disease is endemic worldwide. Affected cattle will produce poorly, thereby causing serious economic losses in the cattle industry, for example, exceeding an estimated $3.8 million annually in Canada [67]. Therefore, prevention of eimeriosis is more important than cure, which can reduce subclinical production losses and reduce the risk of environmental pollution [36].

A study of detection of *Eimeria* oocysts in Chinese dairy farms has shown that 39 of 43 dairy farms had *Eimeria* infection, the average prevalence was 90.7%, and the prevalence of samples in each region ranged from 24.2% to 37.2% [42]. However, there has been no overall systematic estimate of the prevalence of bovine *Eimeria*. In 2017, Sibhat et al. [71] conducted a quantitative review of bovine tuberculosis in Ethiopia by studying 56 qualified research results. The results showed that pooled prevalence of bovine tuberculosis in Ethiopia is about 5.8%, and it proved that intensive cattle husbandry was associated with increased prevalence. The results of the study provided favorable data for the prevention and control of Ethiopian bovine tuberculosis. In 2018, Ran et al. [66] analyzed the occurrence of Bovine viral diarrhea virus (BVDV) seroprevalence in dairy cattle in China by analyzing 41 eligible research results published from March 2003 to March 2018. The results showed that the pooled prevalence of BVDV in dairy cattle in China was approximate 53%. This research was conducive to prevention of BVDV infection in China. Therefore, to understand factors affecting the prevalence of bovine *Eimeria* in China and help reduce the economic loss caused by *Eimeria*, we conducted the first review and meta-analysis of the prevalence of bovine *Eimeria* in this country.

## Materials and methods

### Search strategy and selection criteria

In our systematic review and meta-analysis, as of February 29, 2020, we used Chinese and English to search systematically in five databases: PubMed, ScienceDirect, China National Knowledge Infrastructure (CNKI), Chongqing VIP, and Wanfang. In ScienceDirect, we used “Eimeriidae”, “cattle”, and “China” as keywords. In PubMed, first we used MeSH Terms to search for “Eimeriida”, the Entry terms obtained are “Eimeriidas” and “Eimeriidae”, and the subject words and free words are connected by “OR”. The retrieved search formula is “(“Eimeriida”[Mesh] OR Eimeriidas OR Eimeriidae)”. In the same way, we search for “Cattle”[Mesh] and “China”[Mesh] in turn, and the retrieval formulas obtained are: (“Cattle”[Mesh] OR Bos indicus OR Zebu OR Zebus OR Bos taurus OR Cow, Domestic OR Cows, Domestic OR Domestic Cow OR Domestic Cows OR Bos grunniens OR Yak OR Yaks); “China”[Mesh]) OR People’s Republic of China) OR Mainland China) OR Manchuria) OR Sinkiang) OR Inner Mongolia). The Boolean operator “AND” is used to connect the three search queries. The final search formula is “(“Eimeriida”[Mesh]) OR Eimeriidas) OR Eimeriidae)” AND (“Cattle”[Mesh] OR Bos indicus OR Zebu OR Zebus OR Bos taurus OR Cow, Domestic OR Cows, Domestic OR Domestic Cow OR Domestic Cows OR Bos grunniens OR Yak OR Yaks) AND (“China”[Mesh] OR People’s Republic of China OR Mainland China OR Manchuria OR Sinkiang OR Inner Mongolia)”. In the VIP Chinese Journal Databases, the search strategy set was “title or keywords: coccidiosis AND cattle”. In CNKI and Wanfang Data, the search strategy was “theme: coccidiosis AND cattle”. In the three Chinese databases, all the retrieval processes included fuzzy searches and synonym expansion. We neither contacted the authors of original studies for additional information nor identified related unpublished data. Endnote X9 was used to edit the articles retrieved.

After removing duplicates, we selected each article based on the title and abstract. Then we applied the following inclusion criteria: (1) the purpose of the study was to check the prevalence of *Eimeria* in cattle; (2) the study provided the total number of cattle tested and prevalence; (3) each sample was from one type of cattle (not a mixed sample); (4) the study sample size was greater than 30; and (5) the study design was cross-sectional. Articles that did not meet these criteria were removed.

### Data extraction and quality assessment

Four authors (CYS, BYM, ZYC, and YY) extracted data with standardized data collection forms to identify eligible studies [80]. Any disagreement between the authors or uncertainty about a study was further evaluated by an additional author (QLG). From all the collected studies, we extracted the following information: first author, publication year, sampling year, geographical region of study, location of study, age, *Eimeria* species, species of cattle, detection methods, feeding model, total number of cattle, and number of eimeriosis seropositive cattle. The data collection form is presented in Table 1. The quality of eligible publications was estimated based on criteria derived from the Grading of Recommendations Assessment, Development and Evaluation method [28]. In short, 1 point was awarded for each of the following items: random sampling, the sampling was random, the detection method and sampling method were each described in detail, and the sampling time was clear. A score of 4 or 5 points was deemed high quality; 2 or 3 points, moderate quality; and 0 or 1, low quality.

### Statistical analysis

The pooled prevalence of bovine *Eimeria* based on numerous studies was calculated by meta-analysis. A high chance of heterogeneity in the included studies was presupposed; by using R software for analysis, the results were linear distributions with *W*-values greater than 0.9. Thus, a random-effects model was employed to calculate and prepare forest plots using Stata 12 software (Stata Corp., College Station, TX, USA) [62]. Heterogeneity was anticipated, and statistical methods with *I*^2^ and Cochran’s *Q* (represented as *χ*^2^ and *p*-value) statistics were used to assess the variations. The potential sources of heterogeneity were further investigated by subgroup analysis and meta-regression analysis. The investigated factors comprised the geographical region, the sampled year (comparison of studies published before 2000, between 2000 and 2015, and the period since 2015), the age of cattle (comparison of calves before weaning, calves after weaning, growing cattle with finishing cattle), the species of *Eimeria*, season (comparison of spring and summer with autumn and winter), feeding model (comparison of extensive with intensive), the cattle species and detection method (comparison of saturated saline flotation method, saturated saline precipitation method, saturated sucrose flotation method, comprehensive approach, and other method). We performed a sensitivity analysis for the included studies to verify the stability of the results. Publication bias of the studies included in this meta-analysis was statistically examined with Egger’s test and trim and fill analysis using Stata software (version 12.0). The meta-analysis was performed according to the PRISMA guidelines [60].

## Results

### Included studies

**Figure 1:**
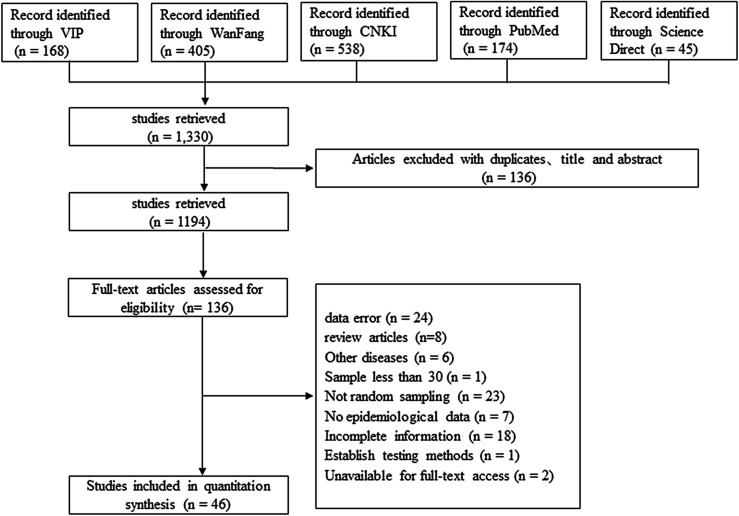
Screening process for eligible articles.

In this study, we searched 1330 articles in five databases, and 46 articles were selected for inclusion in our systematic review and meta-analysis (Fig. 1). We evaluated the quality of each article by region, detection method, sampling year, age of cattle, type of cattle, breeding method and season. Of the 46 articles, 34 were scored as high quality (4 or 5 points), 10 were categorized as medium quality (2 or 3 points), and the remaining two papers were classified as low quality (0 or 1 point) (Table 1).

**Table 1:** Studies included in the analysis.

Study ID	Sampling time	Province	Detection method	Positive samples/total samples (*Coccidia*)	Quality score	Quality level
Central China						
Liu [50]	2012.6–2012.11	Henan	Comprehensive approach	83/460	5	High
Zhao et al. [91]	2011	Henan	Comprehensive approach	134/218	3	Middle
Shi et al. [70]	2009.7–2009.11	Henan	Saturated saline water floating	570/1520	3	Middle
Zhang et al. [97]	UN	Henan	Saturated saline water precipitation	110/503	3	Middle
Chen et al. [12]	2013.3–2013.5	Henan	Comprehensive approach	136/466	5	High
Zhang et al. [98]	1999.03–1999.05	Henan	Saturated saline water precipitation	66/223	3	Middle
Dong et al. [18]	2010	Henan	Saturated saline water precipitation	37/73	5	High
Zhan and Zhao [90]	UN	Hunan	Comprehensive approach	28/32	2	Middle
Eastern China						
Ye [85]	UN	Zhejiang	Others	56/215	3	Middle
Liu et al. [48]	2014.6–2014.12	Shandong	Saturated saline water floating	35/763	3	Middle
Zhao et al. [94]	2005.1–12	Shanghai	Saturated saline water precipitation	269/718	5	High
Lu and Zhang [54]	1990.03–1991.10	Anhui	Saturated saline water floating	233/280	4	High
Li et al. [44]	UN	Anhui	Saturated saline water floating	540/814	4	High
Dong et al. [18]	2010	Shanghai	Saturated saline water precipitation	88/169	5	High
		Shandong				
Dong et al. [19]	2010.11–2011.03	Jiangsu	Others	295/626	4	High
Northern China						
Nie et al. [64]	2015–2017	Inner Mongolia	Comprehensive approach	159/1009	2	Middle
Wang et al. [79]	2007.7–2009.8	Shanxi	Saturated saline water floating	136/1200	3	Middle
Sun [74]	2003.03–2003.04	Inner Mongolia	Comprehensive approach	76/265	4	High
Xu et al. [83]	2009.01–2009.11	Beijing	Saturated saline water floating	1953/3419	4	High
Cao [8]	2015.11–2016.11	Hebei	Saturated saline water floating	107/1020	4	High
Dong et al. [18]	2010	Beijing	Saturated saline water precipitation	89/183	5	High
		Inner Mongolia				
Northwestern China						
Wang [78]	UN	Qinghai	Saturated saline water floating	268/460	2	Middle
Cong [13]	2011.7–2012.8	Shaanxi	Comprehensive approach	65/177	3	Middle
Feng et al. [24]	UN	Shaanxi	Saturated saline water floating	25/49	3	Middle
Zhan [89]	1989.3–1989.5	Qinghai	Saturated saline water floating	37/48	5	High
Zhang et al. [96]	2016.6–2016.8	Xinjiang	Saturated saline water floating	172/524	3	Middle
	2017.1–2017.2					
	2018.1–2018.3					
Ma [55]	2012.9–2014.12	Xinjiang	Saturated saline water floating	48/128	3	Middle
Ma et al. [56]	UN	Xinjiang	Comprehensive approach	75/211	3	Middle
Ni et al. [63]	2014.09	Gansu	Saturated saline water floating	108/234	4	High
Li [46]	UN	Qinghai	Saturated saline water floating	38/50	3	Middle
E L [20]	UN	Qinghai	Saturated saline water floating	156/500	2	Middle
Guo et al. [26]	2014.05–2015.10	Qinghai	Saturated sucrose floating	310/587	4	High
Zhai et al [88]	UN	Shaanxi	Saturated saline water floating	22/48	3	Middle
Jiang [34]	2013.05–2013.06	Xinjiang	Saturated sucrose floating	166/718	4	High
Zhang et al. [95]	2016.08–2016.10	Xinjiang	Saturated sucrose floating	487/1391	5	High
Zhao [92]	UN	Shaanxi	Saturated saline water floating	22/48	4	High
Dong et al. [32]	2010.11–2011.01	Qinghai	Others	113/324	4	High
Southern China						
Liang et al. [47]	2016.4	Guangdong	Comprehensive approach	358/1440	3	Middle
Wei et al. [81]	2013–2014	Guangxi	Comprehensive approach	697/2952	4	High
Wu et al. [82]	2014.1	Guangxi	Comprehensive approach	128/109	2	Middle
Mi et al. [58]	UN	Guangxi	Saturated saline water floating	50/100	2	Middle
Southwestern China						
Yu et al. [87]	UN	Sichuan	Others	10/50	2	Middle
Zhao [14]	1990	Guizhou	Saturated saline water floating	748/1202	3	Middle
Li [43]	2017.8–2017.9	Yuannan	Comprehensive approach	15/44	5	High
Jiang and Zhu [33]	1986.9–1986.12	Sichuan	Others	46/122	2	Middle
Shen et al. [69]	2018.1.20	Sichuan	Comprehensive approach	13/90	5	High
He et al. [31]	2007.05–2008.10	Sichuan	Saturated saline water floating	150/500	3	Middle
Dong et al. [18]	2010	Sichuan	Saturated saline water precipitation	3/10	5	High
Liu [49]	2011.10–2011.11	Yunnan	Saturated saline water floating	37/118	2	Middle

UN*: unclear.

ND*: No data.

Region*: Central China: Henan; Eastern China: Zhejiang, Shandong, Shanghai, Anhu, Hunan Northern China: Inner Mongolia, Shanxi, Beijing, Hebei; Northwestern China: Qinghai, Shaanxi, Xinjiang, Gansu; Southern China: Guangdong, Guangxi; Southwestern China: Sichuan, Guizhou, Yunnan.

### Publication bias

**Figure 2:**
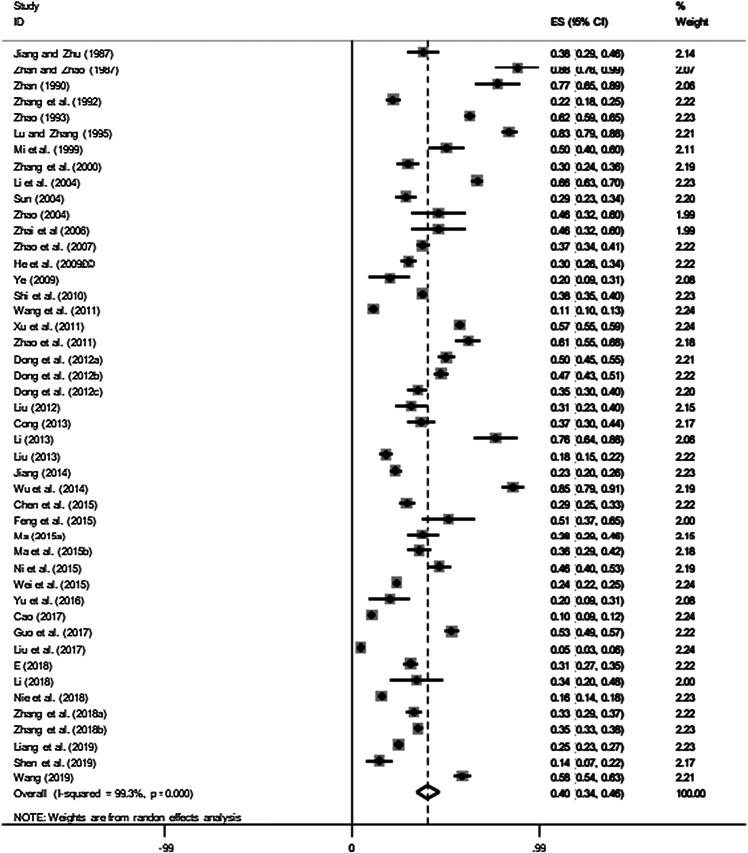
Forest plot of bovine *Eimeria* prevalence among studies conducted in China.

**Figure 3:**
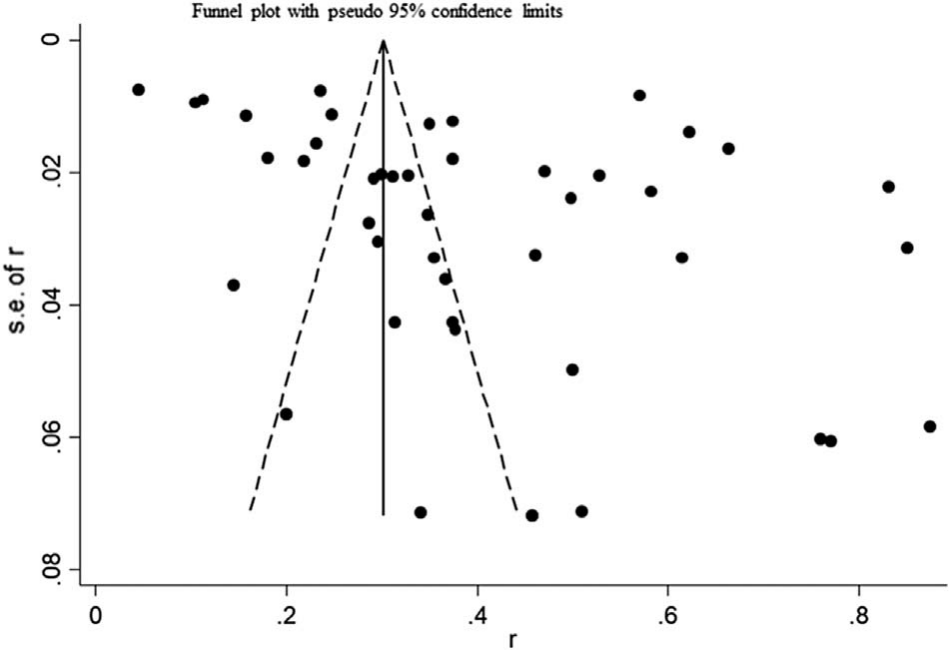
Funnel plot with pseudo 95% confidence limits intervals for the examination o publication bias.

The extent of publication bias in the selected studies was measured and demonstrated by a forest map and funnel plot (Figs. 2 and 3). These data clearly indicated that there were enough publications included to appropriately assess the prevalence of bovine *Eimeria*. The funnel plot did not visually indicate whether there was publication bias, so we conducted Egger’s test and trim and fill analysis to further evaluate the presence of publication bias. Egger’s test result showed *P* = 0.011 (Table S1), and the result of the trim and fill analysis demonstrated that the publication bias disappeared after the addition of two related studies, implying that the studies we included had publication bias or small-study effect bias (Fig. S2, Tables S2 and S3).

### Sensitivity analysis

A sensitivity analysis was also conducted (Fig. S3), in which one study at a time was removed and the others analyzed to estimate whether the results could have been aﬀected markedly by a single study. The results showed that after excluding a certain study, the results of the reorganization analysis are basically consistent with the previous results, so we believe that the results of our meta-analysis are relatively stable and reliable.

### Related factors of bovine *Eimeria* infection in cattle in China

The purpose of our research was to assess the distribution of bovine *Eimeria* in China. The sample distribution we included in the study covered 19 provinces in six regions. Only the northeastern region has no relevant studies. According to our analysis, no statistically significant difference between regions was found (Table 2). Of all the provinces, Hunan had the highest prevalence (87.5%, 95% CI [76.0–99.0]), which may, however, be attributable to small-study bias (Table 3). Data for the included studies were collected between 1986 and 2018; there was a statistically significant difference between the intervals before 2000, between 2000 and 2015, and after 2015, and the prevalence for the first interval was highest, 57.9% (95% CI [39.4–76.4]). There was no statistically significant difference among the different types of cattle, namely: buffalo, dairy cow, scalper, yak, and all others. In our study, calves after weaning (2–12 months old) had the highest prevalence at 37.1% (95% CI [33.6–40.5]). Our study used two sampling seasons: autumn and winter, and spring and summer, among which autumn and winter prevalence was 44.3% (95% CI [13.4–75.3]) and the spring and summer prevalence was 32.9% (95% CI [22.9–42.9]). The prevalence of the saturated saline flotation method in the articles we included was 47.7% (95% CI [35.6–59.8]), higher than that of the saturated saline precipitation method, saturated sucrose water flotation method, comprehensive approach and other methods. The feeding methods in our study include two kinds of breeding methods, extensive and intensive. The prevalence in extensive breeding was 53.9% (95% CI [40.9–67.0]) and that in intensive breeding, 35.2% (95% CI [22.4–48.0]) (Table 2). Pooled prevalence for each of the 17 species of *Eimeria* included, among which *E. bareillyi* had the highest prevalence, is shown in Table 4.

**Table 2:** Pooled prevalence of *Eimeria* in cattle in mainland China.

		No. studies	No. tested	No. positive	% (95% CI*)	Heterogeneity			Univariate meta-regression	
						*χ* ^2^	*p*-value	*I*^2^ (%)	*p*-value	Coefficient (95% CI)*
Region*									0.118	−0.135 (−0.307 to 0.036)
	Central China	8	3495	1164	41.1% (30.7–51.4)	293.04	0.000	97.6%		
	Eastern China	7	3585	1516	45.2% (20.0–70.5)	2247.00	0.000	99.7%		
	Northern China	6	7096	2520	28.6% (9.6–47.6)	1986.08	0.000	99.7%		
	Northwestern China	16	5497	2112	44.2% (37.9–50.5)	336.36	0.000	95.5%		
	Southern China	4	4620	1214	45.5% (28.2–62.9)	385.91	0.000	99.2%		
	Southwestern China	8	2136	1022	32.7% (17.2–48.2)	308.60	0.000	97.7%		
Detection methds									0.102	0.106 (−0.022 to 0.233)
	Saturated saline water precipitation	4	1879	662	34.7% (22.8–46.6)	92.93	0.000	96.8%		
	Saturated saline water floating	19	11,955	5006	47.7% (35.6–59.8)	4874.66	0.000	99.6%		
	Saturated sucrose floating	5	3680	1403	40.3% (28.5–52.1)	228.82	0.000	98.3%		
	Comprehensive approach	13	7492	1948	37.5% (29.4–45.6)	721.09	0.000	98.3%		
	Others	5	1337	520	33.7% (24.1–43.2)	48.38	0.000	91.7%		
Sampling years									0.015	0.233 (0.049 to 0.417)
	Before 2000	5	1875	1130	57.9% (39.4–76.4)	239.45	0.000	98.3%		
	2000–2015	21	15,956	5707	37.2% (28.6–45.9)	3431.15	0.000	99.4%		
	After 2015	6	4509	1152	25.0% (15.1–34.8)	288.45	0.000	98.3%		
Cattle ages*									0.003	−0.153 (−0.253 to −0.053)
	Calves before weaning	6	1024	284	28.9% (22.4–35.4)	24.77	0.000	79.8%		
	Calves after weaning	11	3235	1383	39.9% (34.0–45.7)	233.51	0.000	90.2%		
	Growing cattle	14	2490	1008	38.9% (23.6–54.1)	1286.59	0.000	98.9%		
	Finishing cattle	19	4735	1475	22.7% (14.1–31.2)	1360.35	0.000	98.5%		
Species of cattle										
	Buffalo	6	1907	913	57.4% (37.7–77.0)	351.65	0.000	98.6%	0.052	0.168 (−0.001 to 0.337)
	Dairy cow	25	17,011	6154	38.5% (30.9–46.1)	3060.64	0.000	99.2%		
	Others	3	919	371	43.6% (26.0–61.1)	55.56	0.000	96.4%		
	Scalper	2	995	617	42.5% (−0.9 to 85.8)	56.82	0.000	98.2%		
	Yak	7	2543	1081	45.3% (34.8–55.8)	178.88	0.000	96.6%		
Feeding model									0.115	0. 193 (−0.054 to 0.441)
	Extensive	5	776	388	53.9% (40.9–67.0)	46.48	0.000	91.4%		
	Intensive	10	5691	1540	35.2% (22.4–48.0)	1796.63	0.000	99.5%		
Season*									0.501	0.108 (−0.240 to 0.456)
	Autumn and winter	4	570	267	44.3% (13.4–75.3)	237.14	0.000	98.7%		
	Spring and summer	7	3081	841	32.9% (22.9–42.9)	234.26	0.000	97.4%		
Quality level*									0.228	0.087 (−0.056 to 0.229)
	Middle	10	2692	911	47.0% (30.8–63.1)	2828.64	0.000	99.1%		
	High	34	23,137	8431	38.2% (31.2–45.1)	2771.60	0.000	909.3%		
	Low	2	600	206	40.0% (21.7–58.4)	12.07	0.000	91.7%		
Total		46	26,264	9502	40.0% (34.0–46.0)	6087.31	0.000	99.3%		

CI*: Confidence interval.

Region*: Central China: Henan; Eastern China: Zhejiang, Shandong, Shanghai, Anhu, Hunan Northern China: Inner Mongolia, Shanxi, Beijing, Hebei; Northeastern China: Qinghai, Shaanxi, Xinjiang, Gansu; Southern China: Guangdong, Guangxi; Southwestern China: Sichuan, Guizhou, Yunnan.

Season*: Spring and summer: March through August. Autumn and winter: September through February.

Cattle ages*: Calves before weaning (0 months old to 2 months old), Calves after weaning (2 months old to 12 months old), Growing cattle (12 months old to 24 months old), Finishing cattle (>24 months old).

Quality level *: Low: 0 or 1 points; Middle: 2 or 3 points; High: 4 or 5 points.

**Table 3:** Estimated pooled seroprevalence of *Eimeria* by provincial regions in China.

Province	No. studies	Region	No. tested	No. positive	% Prevalence	% (95% CI)
Anhui	2	Eastern China	1094	773	74.7%	58.2–91.2
Beijing	1	Northern China	3419	1953	57.1%	55.5–58.8
Gansu	1	Northeastern China	234	108	46.2%	39.8–52.5
Guangdong	1	Southern China	1440	358	24.9%	22.6–27.1
Guangxi	3	Southern China	3180	856	52.9%	9.8–95.9
Guizhou	1	Southwestern China	12,062	748	62.2%	59.5–65.0
Hebei	1	Northern China	1020	107	10.5%	8.6–12.4
Henan	6	Central China	3390	1099	32.7%	22.9–42.6
Hunan	1	Central China	32	28	87.5%	76.0–99.0
Inner Mongolia	2	Northern China	1274	235	22.0%	9.3–34.6
Qinghai	6	Northeastern China	1969	922	54.2%	41.6–66.8
Shaanxi	4	Northeastern China	322	134	42.8%	35.9–49.7
Shandong	1	Eastern China	763	35	4.6%	3.1–6.1
Shanghai	2	Eastern China	1344	564	42.3%	32.8–51.7
Shanxi	1	Northern China	1200	136	11.3%	9.5–13.1
Sichuan	4	Southwestern China	762	219	25.7%	16.1–35.3
Xinjiang	5	Northeastern China	2972	948	35.0%	32.5–37.5
Yunan	2	Southwestern China	162	52	32.1%	24.9–39.3
Zhejiang	1	Eastern China	215	56	26.0%	20.2–31.9
Total			25,994	9331	39.9%	33.9–46.0

**Table 4:** Pooled prevalence of different *Eimeria* species in mainland China.

Species of *Coccidia*	No. studies	No. tested	No. positive	% (95% CI*)
*E. auburnensis*	13	5834	672	11.0% (6.6–15.4)
*E. canadensis*	12	5351	594	11.8% (7.4–16.1)
*E. ellips*	14	7021	1154	13.4% (7.8–18.9)
*E. alabamensis*	10	5169	379	8.5% (5.1–12.0)
*E. bareillyi*	1	50	30	60.0% (46.4–73.6)
*E. bovis*	13	6421	1135	23.4% (16.5–30.4)
*E. brasiliensis*	6	3280	179	7.6% (4.1–11.1)
*E. bukidnonensis*	4	1645	180	10.3% (7.7–12.9)
*E. cylindrica*	12	6878	355	6.4% (4.3–8.5)
*E. kwangsiensis*	1	814	28	3.4% (2.2–4.7)
*E. mandela*	1	1520	85	5.6% (4.4–6.7)
*E. pellita*	6	1885	64	3.3% (1.1–5.5)
*E. subspherica*	12	6454	612	12.7% (9.1–16.4)
*E. wyomingensis*	7	4004	231	6.4% (2.5–10.2)
*E. zurnii*	15	7139	1013	17.4% (10.6–12.7)
*E. illinoisensis*	1	118	3	2.5% (−0.3 to 5.3)
*E. stiedai-like*	1	118	1	0.9% (−0.8 to 2.5)

## Discussion and conclusion

Bovine eimeriosis is a parasitic gastrointestinal disease caused by *Eimeria* spp., which is the fifth most important economically and has a major impact on the global cattle industry [41]. To date, 20 species of bovine *Eimeria* have been reported in the world. Oocysts in the environment can be transmitted through the fecal-oral route. Cattle are susceptible to being infected with eimeriosis if they consume contaminated feed, water, or forage [6]. In calves infected with *Eimeria*, fever, anorexia, abdominal pain, dehydration, weakness, and even death may occur. Symptoms in breeding cattle are mainly subclinical, but they can still act as a vector for the protozoa [16]. Because *Eimeria* cause serious economic losses to the cattle industry, we constructed the first meta-analysis to assess the prevalence of bovine eimeriosis in China and potential infection risk factors. The total prevalence of *Eimeria* in Chinese cattle was 40.0%, which was lower than the prevalence in Mexico (60.2%), in North America in cattle (91.7%), and buffalo (81.5%) in three regions of Italy [2, 61]. By contrast, it was higher than the prevalence in the Gwangju area of Korea (10.0%) and in Western Kenya (32.8%) [38, 57].

In the regions we studied, the prevalence in northern China was much lower than those in southern China. This may be caused by the following reasons: first of all, *Eimeria* species oocysts can survive within −30 °C, and the survival time at −5 to 8 °C is much longer than −30 °C [75] The climate in China is very complicated, and the monthly average temperature difference between the north and south in winter can reach about 30 °C [45], so a suitable temperature in the south will be more suitable for the survival of *Eimeria*. Secondly, because a dry climate is not suitable for the survival of *Eimeria* oocysts, infection with bovine *Eimeria* is more serious in the humid southerly regions than in the slightly dry north [17]. In addition, animal husbandry in southern China has developed rapidly, the number of cattle raised is large, and the eimeriosis prevalence will be higher, which is consistent with our research results. However, the difference was not significant (*p* > 0.05), probably because the number of articles in each region we included varies greatly. It is worth noting that we were not able to assess the prevalence of bovine *Eimeria* in the northeast because no studies on bovine *Eimeria* in the northeast were included (Jilin, Liaoning, and Heilongjiang). Importantly, the high prevalence in Hunan Province might be because there was only one article in the province and the total number of samples was only 32. Therefore, in order to more accurately reflect the true prevalence of *Eimeria* in cattle in China, it is recommended to further expand the scope of investigation of *Eimeria* in cattle, increase the sample size of the investigation, and reduce small-study effects bias.

In meta-analysis of rates, the detection method is usually a source of heterogeneity. All the studies we included used traditional fecal testing methods to detect *Eimeria*. The fecal flotation test is the most commonly used technique in clinical laboratory, medical and veterinary medicine for separation of oocysts and eggs. Compared with noncentrifugal flotation methods, centrifugal flotation methods are obviously faster and more efficient [21]. The saturated saline water floating method and saturated sucrose floating method have the highest prevalence in our research, which is consistent with the above conclusions. Research has developed assays that can distinguish *Eimeria* species by polymerase chain reaction (PCR) targeting the species-specific ITS-1 region. Considering sensitivity and reliability, PCR appears to be better than conventional oocyst stool examination and can identify important species of bovine *Eimeria* [35]. Compared with the four flotation options of Brine, Saturated sugar solution, Zinc sulphate solution and Sodium chloride Solution, the Mini-FLOTAC technique using salt/sugar solution is more sensitive and convenient, especially in mixed infections. In addition, this method is suitable for laboratories with limited resources [4]. Traditional fecal testing methods are usually used to identify oocysts. The number of sporangia in the oocysts and the distribution of sporozoites are useful characteristics for distinguishing the genus of *Eimeria*. In addition, it can also be distinguished according to the size, shape and color of the oocysts [21], but they are usually subjective and require significant parasitological expertise and complicated solution preparation, and they are not reliable for species identification [39]. The detection methods used in the articles included in this study are all routine stool detection, which is less convenient, slower, and less accurate than the PCR method. According to the characteristics of *Eimeria* species that can be distinguished by PCR technology, highly pathogenic *Eimeria* can be detected, which provides an effective basis for the prevention and control of *Eimeria*. When the experimental conditions permit, we believe that the PCR method should be selected to detect bovine *Eimeria.*

We found that in the past 32 years, the prevalence of bovine *Eimeria* in China showed a significant downward trend (*p* < 0.05). At the beginning of the reform and opening up, the stock of cattle increased rapidly while little attention was paid to the prevention and control of *Eimeria emmetaria*, so the parasite spread quickly. This is consistent with our research results. In our study, the prevalence of bovine *Eimeria* showed a downward trend year by year, which may have the following reasons: first, since 2000, the Chinese government has successively promulgated policies such as the “Animal Husbandry Law” and “Animal Epidemic Prevention Law” to strengthen support for the development of animal husbandry [11]. Second, in recent years, the level of animal husbandry has developed, and more and more attention has been paid to the prevention and control of bovine *Eimeria*. Commonly used preventive drugs included monensin, amprolium, diclazuril and toltrazuril, and therapeutic drugs included furacilin [51, 65]. This measure has eased the impact of *Eimeria* on the cattle industry. Third, since 2012, the Chinese government has begun to pay more and more attention to environmental pollution caused by animal husbandry: for example, the 12th Five-Year Plan of National Livestock and Poultry Pollution Control issued by the Ministry of Environmental Protection (MEP) and the Ministry of Agriculture (MOA) to strengthen environmental governance [86]. In 2015, the Ministry of Agriculture proposed to implement “Regulations on the Prevention and Control of Pollution from Large-scale Livestock and Poultry Farming,” combined with the pilot project of comprehensive utilization of livestock and poultry manure and other agricultural and rural wastes; strengthen guidance and services; summarize and promote efficient and applicable comprehensive treatment of manure; and provide resources to support the main points of animal husbandry work based on the utilization model (China Ministry of Agriculture 2015). In 2018, the Chinese Ministry of Agriculture proposed to create a new model for the development of animal husbandry, continue to implement county-wide projects to promote the utilization of manure resources, increase capital investment, and expand coverage (China Ministry of Agriculture 2018). The Chinese government has established a rural energy biogas system to manage rural livestock and poultry farming manure, thereby reducing pollution from farming and reducing *Eimeria* prevalence. Therefore, formulating corresponding prevention and control policies may play a positive role in reducing bovine *Eimeria* infection. The above measures have reduced the risk of bovine eimeriosis infection.

There are two main breeding methods in China’s cattle industry: intensive farming model and free-range farming [30]. Intensive, large-scale, standardization is the main strategy for the development of animal husbandry [72]. The transformation of breeding mode in China’s cattle industry reduces the risk of bovine *Eimeria* infection, which is consistent with the results of our study. Our research found that although the point estimate of the intensive farming model is lower than that of extensive, the difference is not significant, and the prevalence of *Eimeria* in cattle under the two farming models is greater than 35%. Intensive farming is denser, and a large amount of manure may not be processed in time, leading to widespread epidemics of infectious diseases [10, 84], which is also the cause of *Eimeria* infection in intensive farming [15]. Further tracing back to the original text, it was found that most studies on intensive farming did not mention the details of manure treatment. At present, some cattle farms in China may not pay attention to the centralized treatment of manure, which has led to uneven quality and prevalence of intensive farming [72]. The “Implementation Opinions on Fighting the Tough Battle for the Prevention and Control of Agricultural Non-point Source Pollution” issued by the Ministry of Agriculture in 2015 requires that the proportion of supporting waste treatment facilities for large-scale livestock and poultry farms (communities) be more than 75% [77]. The “Action Plan for the Zero Growth of Chemical Fertilizer Use by 2020” issued in the same year requires the promotion of the resource utilization of livestock manure and the reduction of chemical fertilizers through organic fertilizer replacement and commitment to livestock manure treatment (Ministry of Agriculture in China, 2015). We found that in articles published after this, the prevalence of *Eimeria* in intensive farming showed a downward trend. We suggest further implementation of high-level intensive farming to increase the utilization of cattle industry resources, while reducing the spread of diseases such as *Eimeria*.

According to reports, *Eimeria* prevalence in cattle of all age groups varies greatly, mainly affecting calves. The prevalence of calves over 6 months old is higher than that of calves 1–6 months old, which may be related to good care and colostrum feeding, especially colostrum can improve the immunity of calves. In addition, 18-month-old cattle are also unlikely to be infected with bovine *Eimeria* due to their highly immunity [3, 22, 25, 37, 57] – this is consistent with our findings. The eimeriosis prevalence of calves before weaning (<2 months) is significantly lower than that of calves after weaning (2–12 months), which may occur because calves acquire antibodies from breast milk [23]. The clinical manifestation of *Eimeria* in calves is characterized by abdominal pain, watery to hemorrhagic diarrhea, fever, and dehydration [37], which have a great impact on the development of the cattle industry. Therefore, based on the results of our research, attention should be paid to the protection of calves to avoid excessive contact between calves and worm-carrying cattle. If conditions permit, feeding calves and adult cattle separately may help prevent the infection of *Eimeria* in calves.

Many breeds of cattle can be infected with *Eimeria*. There are more than 11 common species of *Eimeria* in water buffalo. Among them, *E. bareillyi* is a unique species in buffalo [21], a host animal that is found in warm and humid environments [27], so higher prevalence of *Eimeria* in buffalo may be expected. In our research, the *Eimeria* prevalence of dairy cows is the lowest, which may be due to the high requirements for food safety and the rapid promotion of mechanization, and intelligence and information technology in dairy farming facilities [99]. The dairy farming environment in China is cleaner, which reduces the chance of *Eimeria* infection. In addition, in China, yak and yellow cattle are generally regarded as labor cattle, and they have wider home ranges, so they have more opportunities for contact with infectious *Eimeria* oocysts.

Although season is not the main factor affecting eimeriosis infection in cattle, prevalence vareies with season [73]. A study by Al-Jubory has shown that summer eimeriosis has the lowest prevalence and autumn eimeriosis has a higher prevalence [1] and this may be because the autumn temperature and humidity are more suitable for growth and reproduction. It has also been reported that the lower temperature of *Eimeria* oocysts in winter results in fewer spores and a lower shedding rate [59]; therefore, the prevalence of bovine eimeriosis in winter will be reduced, which is consistent with our research results. We found relatively few articles that recorded the sampling season, and we urge that these data be collected comprehensively to clarify the seasonality of *Eimeria* infection.

Up to eight different *Eimeria* species have arisen in mixed infections [53], though not all species of *Eimeria* are pathogenic. *Eimeria zuernii* and *E. bovis* are considered the most pathogenic [52]. Infection of calves with a large number of oocysts of *E. zuernii* or *E. bovis* may lead to severe diarrhea including blood, intestinal tissue, and fibrin [16]. Lasprilla-Mantilla et al. have also proven this conclusion [40], which is consistent with our research results; however, the difference in our own analysis was not statistically significant. In our study, *E. bareillyi* had the highest prevalence, which may be due to the fact that the total number of samples taken in the study of *E. bareillyi* is only 50 cattle, and the total number of samples is too small, which leads to biased research results. Meanwhile, there are relatively few studies on *E. kwangsiensis*, *E. mandela*, *E. illinoisensis* and *E. stiedai-like*. The prevalence derived from our analysis should be generalized with caution because several included articles did not use random sampling.

The advantages of this research are the wide range of the research, the long time span of the research, the large sample size, and the thorough examination of potential risk factors. There were, however, several limitations to our analysis. Firstly, we identified studies related to bovine *Eimeria* in the selected databases by searching using several different MeSH terms; however, these searches may not have found all the relevant studies. Secondly, some of the factors included in some studies and the sample size are too small, so there may be a small-sample size bias, resulting in unstable outcomes. Finally, the research we included may not be particularly accurate because some of the samples in the included articles were not randomly sampled.

This systematic review and meta-analysis showed that bovine *Eimeria* occurs in most regions of China, and the prevalence has been declining year by year. The age of cattle is one of the main reasons that affects the prevalence of *Eimeria*. To reduce the risk of bovine *Eimeria* infection, farmers should pay special attention to calves, control the density in intensive breeding, keep the breeding environment clean, and strengthen precautions recommended in policy documents. At the same time, it is necessary to develop more convenient, faster, and more accurate methods to detect *Eimeria*.

## Supplementary Material

Supplementary material is available at https://www.parasite-journal.org/10.1051/parasite/2021055/olm *Figure S1*. Egger's publication bias plot. *Figure S2*. Publication bias of studies by Trim ad Fill analysis. *Figure S3*. Sensitive analysis. *Table S1*. Egger’s for publication bias. *Table S2*. Trimming estimator. *Table S3*. Filled meta-analysis. *Table S4*. PRISMA checklist item. *Table S5*. Included studies and quality scores.

## Financial support

This work was funded by the Science and Technology Development Program of Jilin Province (20190304004YY).

## Conflict of interest

The authors declare that they have no competing interests.
