# Supplementary material for: Prevalence and infection risk factors of bovine Eimeria in China: a systematic review and meta-analysis
Source: Parasite. 2021 Aug 10;28:61. doi: 10.1051/parasite/2021055 (PMC8354008; doi:10.1051/parasite/2021055)
Supplement: Supplementary file 1 — Figure S1. Egger's publication bias plot. [file parasite-28-61-s1.pdf]

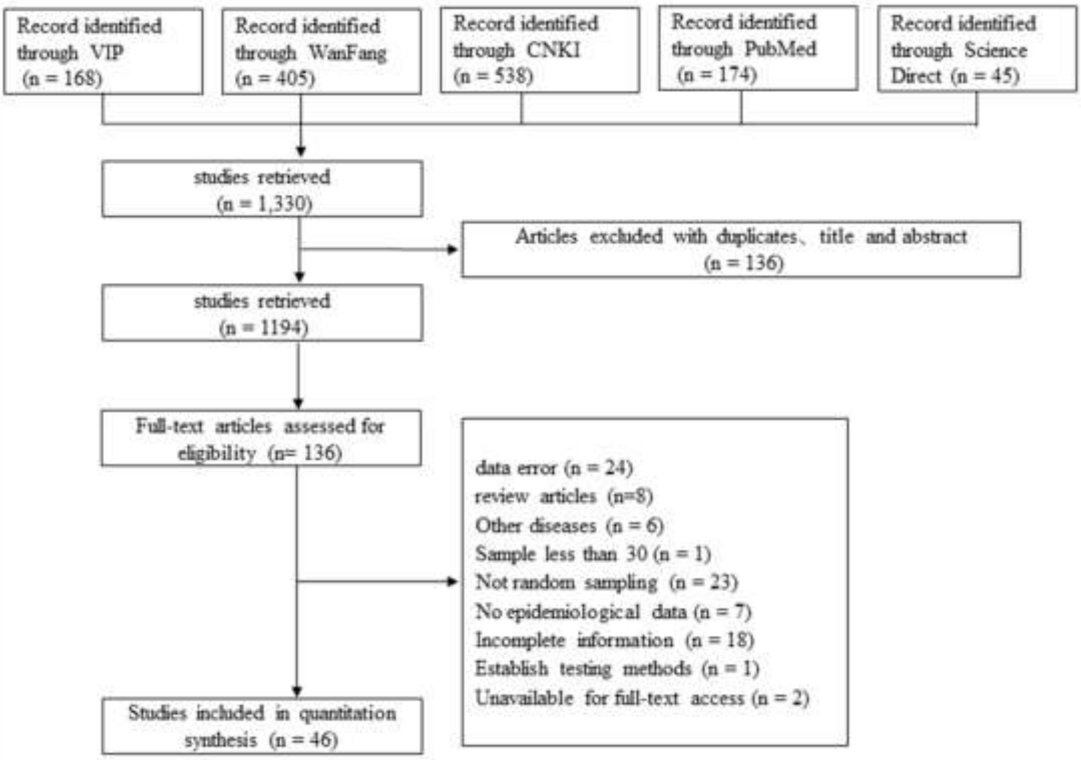

Figure 1 Screening process for eligible articles

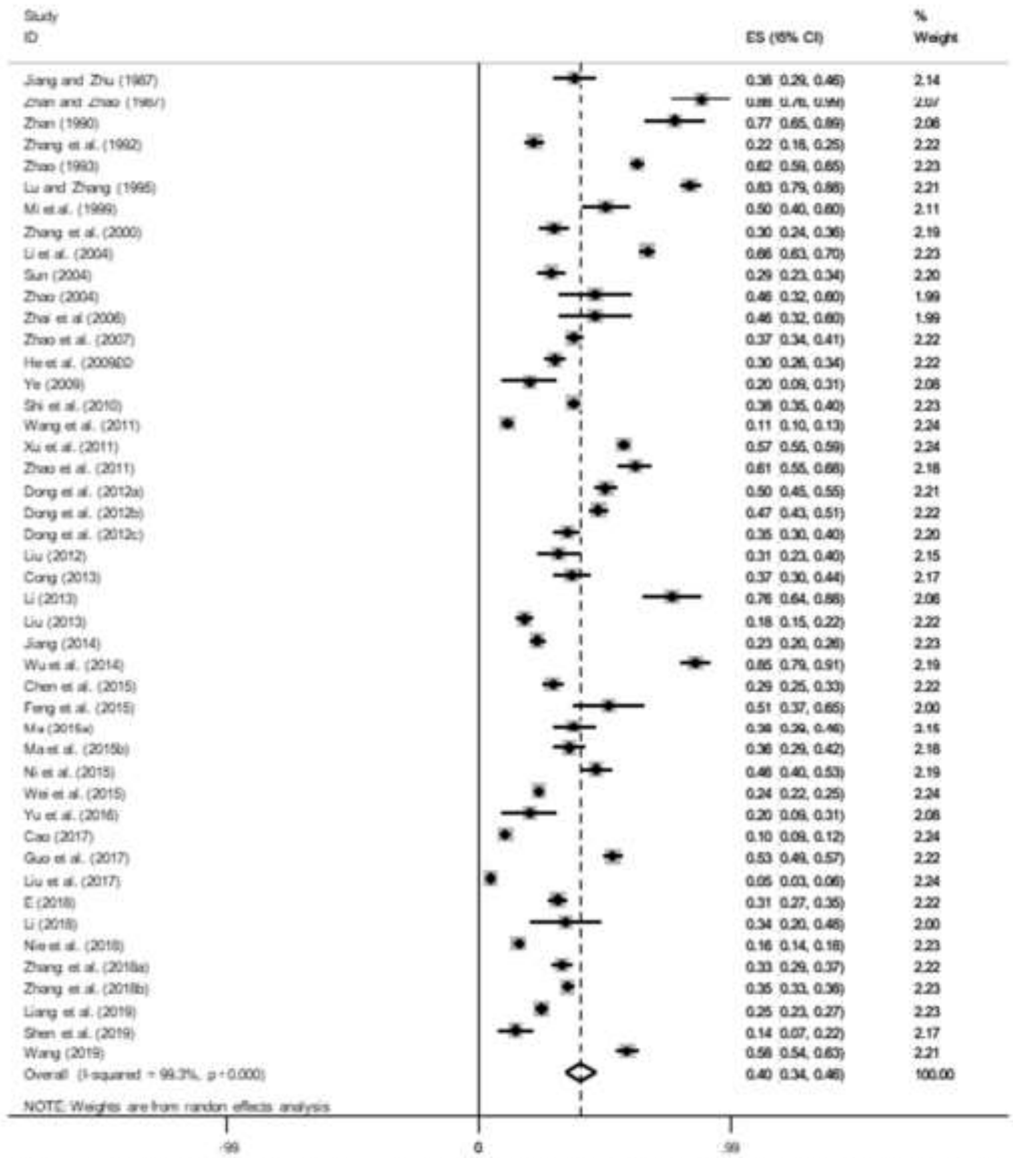

Figure 2 Forest plot of bovine *Eimeria* prevalence among studies conducted in China

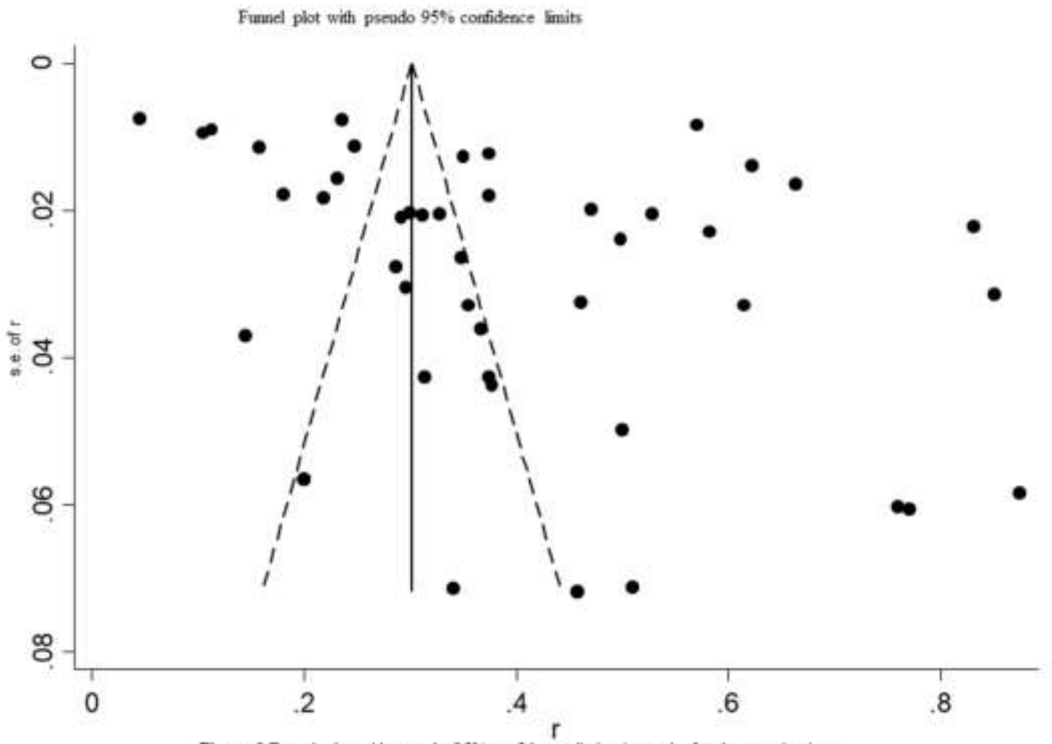

Figure 3 Funnel plot with pseudo 95% confidence limits intervals for the examination of publication bias.

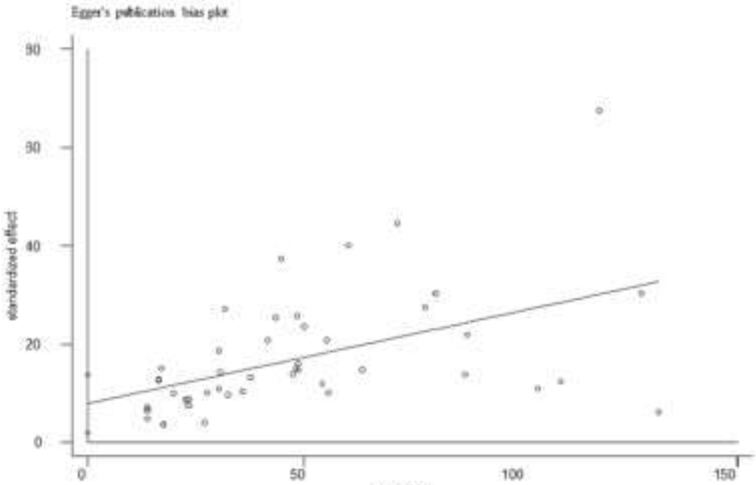

Figure S1 Funnel plot with trim and analysis of the publication bias

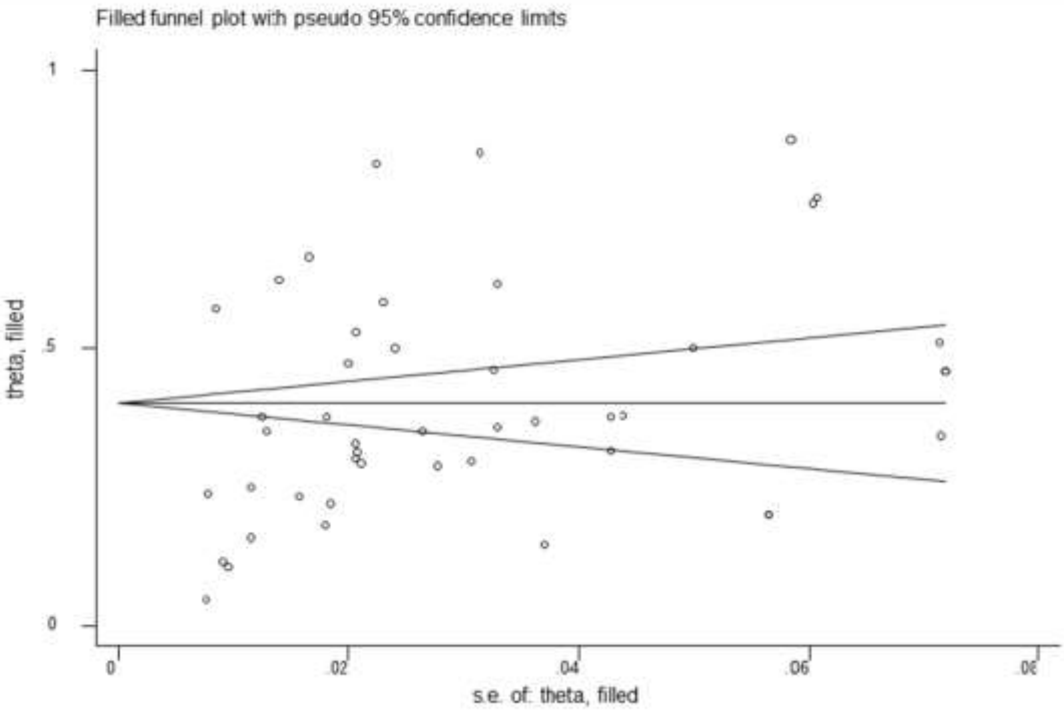

Figure S2 Publication bias of studies by Trim and Fill Analysis

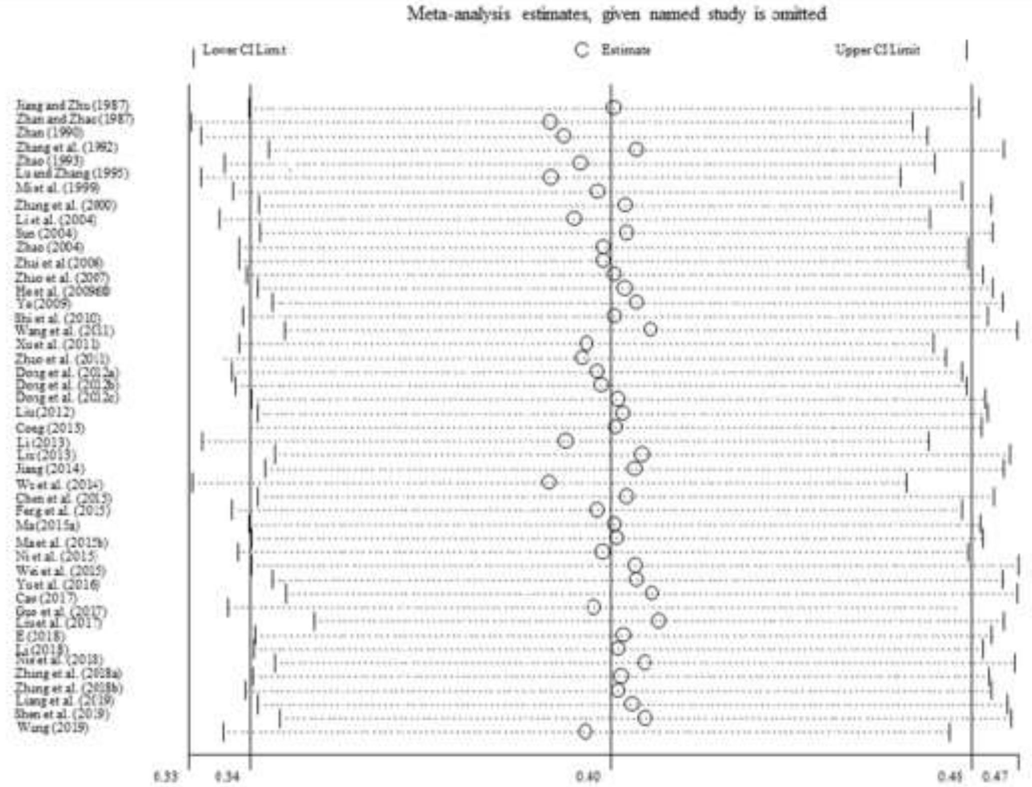

Figure S3 Sensitivity analysis
