# Supplementary material for: Prevalence and infection risk factors of bovine Eimeria in China: a systematic review and meta-analysis
Source: Parasite. 2021 Aug 10;28:61. doi: 10.1051/parasite/2021055 (PMC8354008; doi:10.1051/parasite/2021055)
Supplement: Supplementary file 2 — Table S1. Egger’s for publication bias. [file parasite-28-61-s2.pdf]

Table S1 Egger's for Publication Bias

Egger's test

Std-Eff	Coef.	Std. Err.	t	P>|t|	[95% Conf.Interval]	
**slope**	0.188205	0.0508103	3.70	0.001	0.0858036	0.2
**bias**	7.83699	2.935798	2.67	0.011	1.920278	1

Table S2 Trimming estimator

Trimming estimator: Linear

Meta-analysis type: Random effects model

iteration	estimate	Tn	# to trim	diff
**1**	0.400	509	0	1081
**2**	0.400	509	0	0

Note: no trimming performed; data unchanged.

Table S3 Filled Meta-analysis

Filled

Meta-analysis

**Method**	**Pooled** Est	**95%** Lower	**CI** Upper	**P>|t|** z_value	**Asymptotic** p_value		**No. of** studies
**Fixed**	0.302	0.297	0.307	118.215	0.000	46	
**Random**	0.400	0.340	0.460	13.086	0.000		

Test for heterogeneity: Q=6087.312 on 45 degrees of freedom (p=0.000)

Moment-based estimate of between studies variance=0.042

Table S4. PRISMA Checklist item.

Title	1	Meta-analysis of seroprevalence of Toxoplasma gondii in goats in China from 2010 to 2020	1
**ABSTRACT**			
Structured summary	2	Provide a structured summary including, as applicable: background; objectives; data sources; study eligibility criteria, participants, and interventions; study appraisal and synthesis methods; results; limitations; conclusions and implications of key findings; systematic review registration number.	3
**INTRODUCTION**			
Rationale	3	Describe the rationale for the review in the context of what is already known.	3-4
Objectives	4	Provide an explicit statement of questions being addressed with reference to participants, interventions, comparisons, outcomes, and study design (PICOS).	4-5
**METHODS**			
Protocol and registration	5	Indicate if a review protocol exists, if and where it can be accessed (e.g., Web address), and, if available, provide registration information including registration number.	4-5
Eligibility criteria	6	Specify study characteristics (e.g., PICOS, length of follow-up) and report characteristics (e.g., years considered, language, publication status) used as criteria for eligibility, giving rationale.	4-5
Information sources	7	Describe all information sources (e.g., databases with dates of coverage, contact with study authors to identify additional studies) in the search and date last searched.	4-5 Table S5
Search	8	Present full electronic search strategy for at least one database, including any limits used, such that it could be repeated.	4-5
Study selection	9	State the process for selecting studies (i.e., screening, eligibility, included in systematic review, and, if applicable, included in the meta-analysis).	4-5
Data collection process	10	Describe method of data extraction from reports (e.g., piloted forms, independently, in duplicate) and any processes for obtaining and confirming data from investigators.	4-5
Data items	11	List and define all variables for which data were sought (e.g., PICOS, funding sources) and any assumptions and simplifications made.	4-5

Risk of bias in individual studies	12	Describe methods used for assessing risk of bias of individual studies (including specification of whether this was done at the study or outcome level), and how this information is to be used in any data synthesis.	4-5
Summary measures	13	State the principal summary measures (e.g., risk ratio, difference in means).	4-5
Synthesis of results	14	Describe the methods of handling data and combining results of studies, if done, including measures of consistency (e.g., I^2) for each meta-analysis.	4-5
Risk of bias across studies	15	Specify any assessment of risk of bias that may affect the cumulative evidence (e.g., publication bias, selective reporting within studies).	4-5
Additional analyses	16	Describe methods of additional analyses (e.g., sensitivity or subgroup analyses, meta-regression), if done, indicating which were pre-specified.	4-5
**RESULTS**			
Study selection	17	Give numbers of studies screened, assessed for eligibility, and included in the review, with reasons for exclusions at each stage, ideally with a flow diagram.	5-6, Figure 1
Study characteristics	18	For each study, present characteristics for which data were extracted (e.g., study size, PICOS, follow-up period) and provide the citations.	6
Risk of bias within studies	19	Present data on risk of bias of each study and, if available, any outcome level assessment (see item 12).	6, Figure 4, Figure S1 and Figure S2
Results of individual studies	20	For all outcomes considered (benefits or harms), present, for each study: (a) simple summary data for each intervention group (b) effect estimates and confidence intervals, ideally with a forest plot.	6, Figure 2
Synthesis of results	21	Present results of each meta-analysis done, including confidence intervals and measures of consistency.	6, Figure 2
Risk of bias across studies	22	Present results of any assessment of risk of bias across studies (see Item 15).	6, Figure 4, Figure 5, Figure S1, Figure S2
Additional analysis	23	Give results of additional analyses, if done (e.g., sensitivity or subgroup analyses, meta-regression [see Item 16]).	6, Figure S3, Table S1, Table S2 and Table S3
**DISCUSSION**			

Summary of evidence	24	Summarize the main findings including the strength of evidence for each main outcome; consider their relevance to key groups (e.g., healthcare providers, users, and policy makers).	7-8
Limitations	25	Discuss limitations at study and outcome level (e.g., risk of bias), and at review-level (e.g., incomplete retrieval of identified research, reporting bias).	7-10
Conclusions	26	Provide a general interpretation of the results in the context of other evidence, and implications for future research.	7-10
**FUNDING**			
Funding	27	Describe sources of funding for the systematic review and other support (e.g., supply of data); role of founders for the systematic review.	11

From: Moher D, Liberati A, Tetzlaff J, Altman DG, The PRISMA Group (2009). Preferred Reporting Items for Systematic Reviews and Meta-Analyses: The PRISMA Statement. PLoS Med 6(6): e1000097. doi:10.1371/journal.pmed1000097

For more information, visit: **www.prisma-statement.org**.

Table S5. Included studies and quality scores**.**

	Reference ID	No. tested	No. positive	Prevalence	Study design	random sampling	Detection method detailedly or not	Sampled method detailedly or not	Sampled time clearly or not	Three or more risk factors or not	Score	Study Quality
1	Jiang and Zhu (1987)	122	46	0.377	Cross sectional	0	1	0	1	0	2	middle
2	Zhan and Zhao (1987)	32	28	0.875	Cross sectional	0	1	1	0	0	2	middle
3	Zhan (1990)	48	37	0.771	Cross sectional	1	1	1	1	1	5	high
4	Zhang et al. (1992)	503	110	0.219	Cross sectional	1	1	1	0	1	4	high
5	Zhao (1993)	1202	748	0.622	Cross sectional	0	1	1	1	1	4	high
6	Lu and Zhang (1995)	280	233	0.832	Cross sectional	0	1	1	1	1	4	high
7	Mi et al. (1999)	100	50	0.500	Cross sectional	0	1	0	0	0	1	low
8	Zhang et al. (2000)	223	66	0.296	Cross sectional	0	1	0	1	0	2	middle
9	li et al. (2004)	814	540	0.663	Cross sectional	1	1	1	0	1	4	high
10	sun (2004)	265	76	0.287	Cross sectional	0	1	1	1	1	4	high
11	Zhao (2004)	48	22	0.458	Cross sectional	1	1	1	0	1	4	high
12	zhai et al (2006)	48	22	0.458	Cross sectional	1	1	1	0	1	4	high
13	Zhao et al. (2007)	718	269	0.375	Cross sectional	1	1	1	1	1	5	high
14	Ye (2009)	215	56	0.260	Cross sectional	1	1	1	0	1	4	high
15	He et al. (2009)	500	150	0.300	Cross sectional	0	1	0	1	0	2	middle
16	Shi et al. (2010)	1520	570	0.375	Cross sectional	0	1	1	1	1	4	high
17	Zhao et al. (2011)	218	134	0.615	Cross sectional	0	1	1	1	1	4	high
18	Wang et al. (2011)	1200	136	0.113	Cross sectional	0	1	1	1	1	4	high
19	Xu et al. (2011)	3419	1953	0.571	Cross sectional	0	1	1	1	1	4	high
20	Dong et al. (2012a)	435	217	0.499	Cross sectional	1	1	1	1	1	5	high
21	Dong et al. (2012b)	626	295	0.471	Cross sectional	0	1	1	1	1	4	high
22	Dong et al. (2012c)	324	113	0.349	Cross sectional	0	1	1	1	1	4	high
23	Liu (2012)	118	37	0.314	Cross sectional	0	1	0	1	0	2	middle
24	Liu (2013)	460	83	0.180	Cross sectional	1	1	1	1	1	5	high
25	Cong (2013)	177	65	0.367	Cross sectional	0	1	1	1	1	4	high

26	Li (2013)	50	38	0.76	Cross sectional	0	1	1	0	0	2	middle
27	Wu et al. (2014)	128	109	0.852	Cross sectional	0	1	0	1	0	2	middle
28	Jiang (2014)	718	166	0.231	Cross sectional	0	1	1	1	1	4	high
29	Wei et al. (2015)	2952	697	0.236	Cross sectional	1	1	1	1	1	5	high
30	Chen et al. (2015)	466	136	0.292	Cross sectional	1	1	1	1	1	5	high
31	Feng et al. (2015)	49	25	0.510	Cross sectional	1	1	1	0	1	4	high
32	Ma (2015a)	128	48	0.375	Cross sectional	1	1	0	1	1	4	high
33	Ma et al. (2015b)	211	75	0.355	Cross sectional	1	1	1	0	1	4	high
34	Ni et al. (2015)	234	108	0.462	Cross sectional	0	1	1	1	1	4	high
35	Yu et al. (2016)	50	10	0.200	Cross sectional	1	1	0	0	0	2	middle
36	Liu et al. (2017)	763	35	0.046	Cross sectional	0	1	1	1	1	4	high
37	Cao (2017)	1020	107	0.105	Cross sectional	0	1	1	1	1	4	high
38	Guo et al. (2017)	587	310	0.528	Cross sectional	0	1	1	1	1	4	high
39	Li (2018)	44	15	0.341	Cross sectional	1	1	1	1	1	5	high
40	Nie et al. (2018)	1009	159	0.158	Cross sectional	0	1	0	1	0	2	middle
41	Zhang et al. (2018a)	524	172	0.328	Cross sectional	0	1	1	1	1	4	high
42	E (2018)	500	156	0.312	Cross sectional	0	1	0	0	0	1	low
43	Zhang et al. (2018b)	1391	487	0.350	Cross sectional	1	1	1	1	1	5	high
44	Liang et al. (2019)	1440	358	0.249	Cross sectional	0	1	1	1	1	4	high
45	Wang (2019)	460	268	0.583	Cross sectional	0	1	1	0	0	2	middle
46	Shen et al. (2019)	90	13	0.144	Cross sectional	1	1	1	1	1	5	high

Y*: Yes; N*: No.

References

1. AKANDE FA, PHILIP AM. 2020. Diagnosis of bovine gastrointestinal parasites: comparison of different techniques and different solutions.

2. Al-Jubory QJA. 2016. Prevalence of Eimeriaparasite in cattle in Al-Najaf province and its relation to risk factors: age, gender and season. Euphrates Journal of Agriculture Science, 8(3), 9–18.

3. Alemayehu A, Nuru M, Belina T, Mekibib B, Desta T, Tesfaye DJJoVM, Health A. 2013. Prevalence of bovine coccidia in Kombolcha district of South Wollo, Ethiopia. Journal of Veterinary Medicine, 5(2), 41–45.

4. Anjos AVD, De MEVCS, Souza MNED, Dias MA. 2011. Frequency of species of the Genus Eimeria in naturally infected cattle in Southern Bahia, Northeast Brazil. Revista Brasilra De Parasitologia Veterinária, 20(1), 78–81.

5. Bangoura B, Bardsley KD. 2020. Ruminant Coccidiosis. Veterinary Clinics: Food Animal Practice, 36(1), 187–203.

6. Bueno CL, Eduardo NJO, Azeredo BTDS, Monteiro CLF, Dos SJB, Beltrán ZDM, Assis CASD, Cayeiro CB, Lino BDGA, Almeida BFDJRBDPV. 2018. Eimeria species in dairy and beef cattle of different ages in Goiás state, Brazil. Revista Brasileira De Parasitologia Veterinária, 27(2), 169.

7. Cardim ST, Seixas M, Tabacow VBD, Taroda A, Carneiro PG, Martins TA, Barros LD, Minutti AF, Chryssafidis AL, Vidotto O, Garcia JL. 2018. Prevalence of Eimeria spp. in calves from dairy farms in northern Paraná state, Brazil. Rev Bras Parasitol Vet, Rev Bras Parasitol Vet, 27(1), 119-123.

8. Das M, Deka D, Sarmah P, Islam S, Sarma S. 2015. Diversity of Eimeria spp. in dairy cattle of Guwahati, Assam, India. Veterinary world, 8(8), 941.

9. Daugschies A, Najdrowski M. 2005. Eimeriosis in cattle: current understanding. Journal of Veterinary Medicine, Series B, 52(10), 417-427.

10. Dong H, Zhao QP, Han HY, Jiang LL, Zhu ShS, Li T, Kong ChL, Huang B. 2012. Prevalence of Coccidial Infection in Dairy Cattle in Shanghai, China. Journal of Parasitology, 98(5), 963.

11. Mitchell E S E, Smith R P, Ellis-Iversen J. 2012. Husbandry risk factors associated with subclinical coccidiosis in young cattle. Veterinary Journal.

12. Ekawasti F, Nurcahyo W, Wardhana AH, Shibahara T, Tokoro M, Sasai K, Matsubayashi M. 2019. Molecular characterization of highly pathogenic Eimeria species among beef cattle on Java Island, Indonesia. Parasitology international, 72, 101927.

13. El-Alfy E, Abbas I, Al-Kappany Y, Al-Araby M, Abu-Elwafa SA, Dubey JP. 2019. Prevalence of Eimeria Species in Water Buffaloes (Bubalus bubalis) from

Egypt and First Report of Eimeria bareillyi Oocysts. Journal of Parasitology, 105(5), 748-754.

14. Faber JE, Kollmann D, Heise A, Bauer C, Failing K, Bürger HJ, Zahner H. 2002. Eimeria infections in cows in the periparturient phase and their calves: oocyst excretion and levels of specific serum and colostrum antibodies. Veterinary Parasitology, 104(1), 1-17.

15. Gebeyehu B, Kebede E, Kifleyohannes T, Abebe N, Kumar N. 2018. Prevalence of calf coccidiosis in Mekelle, northern Ethiopia. Ethiopian Veterinary Journal, 22(2).

16. Gupta A, Singh NK, Singh H, Rath SS. 2016. Assessment of risk factors associated with prevalence of coccidiosis in dairy animals of Punjab. Journal of Parasitic Diseases, 40(4), 1359.

17. Guyatt GH, Oxman AD, Vist GE, Kunz R, Falck-Ytter Y, Alonso-Coello P, Schunemann HJJB. 2008. GRADE: an emerging consensus on rating quality of evidence and strength of recommendations. 336(7650), 924-926.

18. Liu ZH, Zhang YZ. 2019. Diagnosis and control of bovine coccidiosis. Contemporary livestock and poultry breeding industry.

19. Hamid PH, Kristianingrum YP, Prastowo S. 2019. Bovine coccidiosis cases of beef and dairy cattle in Indonesia. Veterinary Parasitology: Regional Studies and Reports, Veterinary Parasitology: Regional Studies and Reports, 17, 100298.

20. Han XP, Hubbert B, Hubbert M, Reinhardt CD. 2016. Overview of the beef cattle industry in China: The widening deficit between demand and output in a vicious circle. Journal of Fisheries & Livestock Production, 2016.

21. Dejene A, Deneke Y, Ibrahim N. 2016. Prevalence and associated risk factors of calf coccidiosis in and around Asela Town, Southeast Ethiopia. Journal of Natural Sciences Research, 6(3).

22. Chai HG. 2019. Occurrence characteristics and prevention and control measures of cattle disease. Chinese Journal of Animal Husbandry and Veterinary Medicine.

23. Kawahara F, Zhang G, Mingala CN, Yu T, Nunoya T. 2010. Genetic analysis and development of species-specific PCR assays based on ITS-1 region of rRNA in

bovine Eimeria parasites. Veterinary Parasitology, 174(1-2), 49-57.

24. Keeton STN, Navarre CB. 2018. Coccidiosis in large and small ruminants. Veterinary Clinics: Food Animal Practice, 34(1), 201-208.

25. Kim H-C, Choe C, Kim S, Chae J-S, Yu D-H, Park J, Park B-K, Choi K-S. 2018. Epidemiological survey on Eimeria spp. associated with diarrhea in pre-weaned native Korean calves. The Korean journal of parasitology, 56(6), 619.

26. Koh B-R-D, Kim H-J, Oh A, Jung B-R, Park J-S, Lee J-G, Na H-M, Kim Y-H. 2019. Prevalence of enteropathogens in the feces from diarrheic Korean native cattle in Gwangju area, Korea. Korean Journal of Veterinary Service, 42(2), 93-112.

27. Lalonde LF, Gajadhar AA. 2011. Detection and differentiation of coccidian oocysts by real-time PCR and melting curve analysis. Journal of Parasitology, 97(4), 725-730.

28. Lasprilla-Mantilla MI, Wagner V, Pena J, Frechette A, Thivierge K, Dufour S, Fernandez-Prada C. 2019. Effects of recycled manure solids bedding on the spread of gastrointestinal parasites in the environment of dairies and milk. Journal of dairy science, 102(12), 11308-11316.

29. Lee S-H, Kim H-Y, Lee H, Kim JW, Lee Y-R, Chae MJ, Oh S-I, Kim JH, Rhee MH, Kwon O-D. 2018. Eimeria species in cattle with diarrhoea in the Republic of Korea regarding age, season and nature of diarrhoea. Veterinary Record, vetrec-2017-104600.

30. Li H, Guo YJ, Liu C, Pan BL, Du QJ. 2016. Epidemiological investigation of cow coccidiosis in some areas of my country. Chinese Journal of Animal Science, 52(14), 35-38.

31. Li QP, Ding YH. 2004. Multi-year simulation and performance verification of regional climate models on East Asian monsoon and precipitation in China. Acta Meteorologica Sinica, 62(2), 140-153.

32. Li XZ, Yan CG, Zan LS. 2018. Current situation and future prospects for beef production in China — A review. Asian-Australasian Journal of Animal Sciences, 31(7), 984-991.

33. López-Osorio S, Silva LM, Taubert A, Chaparro-Gutiérrez JJ, Hermosilla CR. 2018. Concomitant in vitro development of Eimeria zuernii-and Eimeria

bovis-macromeronts in primary host endothelial cells. Parasitology International, 67(6), 742-750.

34. Lopez-Osorio S, Villar D, Failing K, Taubert A, Hermosilla C, Chaparro-Gutierrez JJ. 2020. Epidemiological survey and risk factor analysis on Eimeria infections in calves and young cattle up to 1 year old in Colombia. Parasitology Research, 119(1), 255-266.

35. Makau D, Gitau G, Muchemi G, Thomas L, Cook EA, Wardrop N, Fèvre EM, de Glanville WA. 2017. Environmental predictors of bovine Eimeria infection in western Kenya. Tropical Animal Health & Production, 49(2), 409-416.

36. This publication. 2015. Ministry of Agriculture: In 2020, the manure treatment facilities of farms will reach more than 75%. Jiangxi Journal of Animal Husbandry & Veterinary Medicine, 000(5), 49.

37. Moher D, Shamseer L, Clarke M, Ghersi D, Liberati A, Petticrew M, Shekelle P, Stewart LA. 2015. Preferred reporting items for systematic review and meta-analysis protocols (PRISMA-P) 2015 statement. Systematic reviews, 4(1), 1.

38. Ni HB, Gong QL, Zhao Q, Li XY, Zhang XX. 2020. Prevalence of Haemophilus parasuis"Glaesserella parasuis" in pigs in China: A systematic review and meta-analysis. Preventive Veterinary Medicine, 182, 105083.

39. Chen C. 2011. Retrospect and Prospect of my country's Animal Husbandry Development Since the New Century. Agricultural Outlook, 07(6), 39-42.

40. Rehman TU, Khan MN, Sajid MS, Rao ZA, Arshad M, Iqbal Z, Iqbal A. 2011. Epidemiology of Eimeria and associated risk factors in cattle of district Toba Tek Singh, Pakistan. The Indian journal of animal Sciences, 82(5), 1171-1177.

41. Zheng GS, Shi ZX, Teng GH. 2019. Research progress on equipment technology of dairy farming facilities in China. Chinese Journal of Animal Science. 55(07), 169-174.

42. Sara LO, Silva LMR, Anja T, Chaparro-Gutiérrez JJ, Hermosilla CR. 2018. Concomitant in vitro development of Eimeria zuernii- and Eimeria bovis-macromeronts in primary host endothelial cells. Parasitology International, 67(6), 742-750.

43. Sims J, Bergström L, Bowman B, Oenema OJSU, Management. 2005. Nutrient management for intensive animal agriculture: policies and practices for

sustainability. SOIL USE AND MANAGEMENT, 21(1), 141-151.

44. Stewart DI, Smith PR, Ellis J. 2008. Eimeria species in cattle on farms in England and Wales. Veterinary Record, 162(15), 482.

45. Svensson C, Uggla A, Pehrson B. 1994. Eimeria alabamensis infection as a cause of diarrhoea in calves at pasture. Veterinary Parasitology, 53(1-2), 33-43.

46. Swarnkar CP, Singh D. 2020. Prevalence of Eimeria species in sheep of arid and semi-arid Rajasthan. Veterinary Parasitology, 26(1), 79-85.

47. Wang W, Gong QL Zeng A, Li MH, Zhao Q, Ni HB. 2020. Prevalence of Cryptosporidium in pigs in China: A systematic review and meta- analysis. Transboundary and Emerging Diseases.

48. Q Y, S KH, Hu T, Ying TY. 2018. Environmental status of livestock and poultry sectors in China under current transformation stage. Science of The Total Environment, 622, 702-709.

49. Xu Y, Li J, Zhang XB, Wang LQ, Xu XB, Xu L, Gong HR, Xie HY, Li FD. 2019. Data integration analysis: Heavy metal pollution in China's large-scale cattle rearing and reduction potential in manure utilization. Veterinary Parasitology, 232, 308-317.
